# ID 82 - Estratégias de Rastreamento de Câncer de Pele Promovidas pelo Projeto Retrate do Hospital de Câncer de Barretos: uma avaliação econômica

**DOI:** 10.5327/2237-9622.2025.v34s1.82

**Published:** 2025-11-25

**Authors:** Fernanda Silva Desidério, Fernanda Silva Desidério, Antônio Carlos Pereira, Raquel Descie Veraldi Leite, Vinicius de Lima Vazquez

**Keywords:** câncer de pele, melanoma, rastreamento, custo-efetividade, teledermatologia

## Abstract

**Introdução:**

O câncer de pele, especialmente o melanoma, constitui um desafio crítico para a saúde pública no Brasil devido ao aumento da incidência e ao impacto econômico e social do diagnóstico tardio. O diagnóstico precoce é essencial para melhorar os resultados clínicos e minimizar os custos associados. Nesse contexto, o Projeto Retrate, desenvolvido no Hospital de Amor (HA) e financiado pelo Programa Nacional de Apoio à Atenção Oncológica (Pronon), visa implementar estratégias inovadoras de rastreamento para câncer de pele, com foco em melanoma. As estratégias incluem capacitação de profissionais da saúde e da estética, campanhas de busca ativa em grupos de risco e desenvolvimento de tecnologias de teledermatologia, como um aplicativo móvel e uma cabine fotográfica para autoatendimento. Este estudo busca avaliar a custo-efetividade das diferentes abordagens de rastreamento propostas pelo Projeto Retrate, com o objetivo de identificar a mais eficiente para o Sistema Único de Saúde (SUS).

**Método:**

A avaliação de custo-efetividade (ACE) será realizada conforme as Diretrizes Metodológicas da Rede Brasileira de Avaliação de Tecnologias em Saúde (Rebrats), comparando diferentes estratégias de rastreamento: direcionado, oportunístico e populacional. O estudo incluirá todos os participantes das estratégias de rastreamento do Projeto Retrate, na região da cidade de Barretos/SP. Serão comparadas as abordagens de triagem de pacientes pela cabine fotográfica e aplicativo de autoatendimento com a triagem de pacientes enviados para teletriagem, decorrente da capacitação de profissionais de saúde do atendimento primário. A análise de custo-efetividade será realizada do ponto de vista do Hospital de Amor, permitindo uma avaliação dos custos e impactos da implementação das estratégias de rastreamento de câncer de pele na instituição, que atende 100% de seus pacientes pelo SUS. O desfecho clínico primário será a identificação precoce de câncer de pele, com ênfase em melanoma. Uma árvore de decisão será usada para representar o fluxo do processo de rastreamento, desde a implementação das abordagens até o diagnóstico, representando graficamente as decisões e desfechos clínicos ao longo do processo de rastreamento, facilitando a análise de custo-efetividade. Serão avaliados número de casos identificados durante o rastreamento, taxa de identificação precoce em comparação aos métodos convencionais, e tempo entre a identificação da lesão e o diagnóstico definitivo.

**Resultados:**

Espera-se que o estudo identifique a estratégia de rastreamento mais custo-efetiva e viável para o diagnóstico precoce de câncer de pele, especialmente melanoma. Resultados preliminares sugerem que a combinação de tecnologias de teledermatologia e campanhas de busca ativa em grupos de risco poderá apresentar o melhor custo-benefício para o SUS, ao reduzir os custos de tratamento e melhorar a sobrevida dos pacientes.

**Conclusão:**

A implementação de programas de rastreamento mais eficazes pode reduzir os custos e aumentar a eficiência dos serviços de saúde no Brasil. A comparação dos resultados com a literatura internacional reforça a relevância do diagnóstico precoce para a otimização de recursos. Este estudo fornecerá subsídios para a formulação de políticas públicas que promovam a sustentabilidade e a eficiência do sistema de saúde, melhorando a qualidade do cuidado ao paciente.

